# A heuristics matrix for evaluating the learnability of safety critical medical systems

**DOI:** 10.1016/j.csbj.2025.09.001

**Published:** 2025-09-11

**Authors:** Jessica Turner, Judy Bowen, Veela Moxham-Bettridge

**Keywords:** Interactive systems, Learnability, Safety-critical devices, Usability, User perspectives, Heuristic evaluation

## Abstract

The increasing use of medical devices in non-clinical settings has elevated the importance of learnability, a key aspect of usability, that is defined as how easily users can learn to operate a system. While learnability is recognised in international usability standards, its varied definitions and inconsistent application emphasise the need for more targeted evaluation methods. This paper addresses this gap by proposing a framework for assessing learnability based on user experience levels and context of use. We define a set of user categories and introduce a learnability matrix to evaluate how different types of users, such as patients, caregivers, and medical professionals, interact with medical technologies. To illustrate the methodology, we perform a case study using the Medtronic MiniMed 770G insulin pump system. Our analysis shows that while primary and secondary users are often well-supported, passive, infrequent or adhoc users may face challenges in learning to use the device effectively in emergency settings. These findings underscore the need for more inclusive design and evaluation approaches that account for multiple user roles, to improve the safety and usability of home-based medical technologies.

## Graphical abstract

**Figure:**
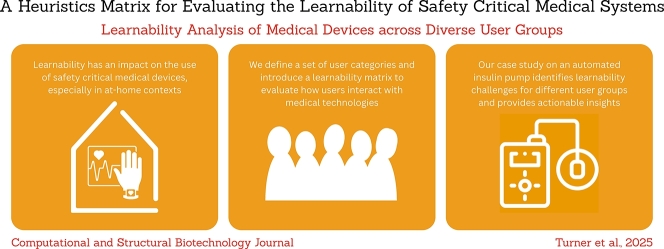

## 1 Introduction

In the process of engineering interactive systems, usability is a key quality metric to ensure that systems are not only functional but are also fit for purpose for their target audience. Quesenbery defines the 5 dimensions of usability as: effective, in that the system helps users reach their goals; efficient, how quickly users can achieve their goals; engaging, the system is satisfying to use; error tolerant, the system not only prevents errors but helps users to recover; and lastly, learnable, how well users can learn to use the system by building on their existing understanding [27]. While each of these elements are essential to ensure that all systems are usable, their importance is amplified for safety-critical contexts, in which errors could lead to harm or even fatalities.

Medical devices are used in healthcare to diagnose, monitor or treat medical conditions. As safety-critical interactive systems, when errors occur the consequences can have serious negative impacts on the end user's health outcomes. For example, pacemakers are a medical device used to help users regulate their heartbeat. Alfayez et al. [1] highlight the benefits of pacemakers and the common challenges with hardware, while Huhn and Bessling [13] emphasise the importance of verifying the functionality of pacemakers' software to ensure reliability and identify hazards before they occur. Ensuring correct functionality and behaviour of medical devices is essential to ensure products work as expected to improve health outcomes, however, if these devices are not learnable by end users, health benefits are significantly reduced.

Learnability has many definitions in literature depending on the end user's experience level. Much like Quesenbery, Unsöld [36] discusses how both ISO 9241-110:2006 and ISO/IEC 25010:20 define learnability as one key aspect of usability but both provide different definitions. The first standard defines learnability as simply “suitable for learning” while the second standard refers to a specified group of people using a system with “effectiveness, efficiency, freedom from risk and satisfaction”. Both standards also refer to a specific context of use. Similarly, Grossman et al. [11] observe three types of learnability definitions based on user experience: *initial learning* in which a novice user can learn how to use a system within a short period of time; *extended learning* in which quality of use improves over time as a user becomes more familiar with a system; and lastly *learning as a function of experience* where a user may have domain knowledge of similar systems but not of the current one in use. More generally, we could define learnability as simply “easy to learn” in the same way usability can be referred to as “easy to use”. However, much like usability, it is evident from these wide range of definitions that learnability is far more complex than this simple definition suggests.

As a key aspect of usability, in this paper we build on these pre-existing definitions to define *learnability* as the ability for a user to learn how to use a system depending on their experience level, domain knowledge and familiarity. That is, we consider learnability as directly linked to the ways (how, when and how often) in which a user interacts with a system, from adhoc or novice users who may not have encountered the system before, to expert users who engage with the system regularly. Note that this differs from usability as it does not include all aspects such as effectiveness, efficiency, engagement, or error tolerance. For example, while an engaging system may increase its likability and usability overall, an engaging system that no one can learn how to use is pointless. This is not to say user engagement is not important, but rather when we consider the domain of medical devices, systems which are difficult to use and unlearnable will have unintended consequences which negatively impact the health of end users.

The Emergency Care Research Institute (ECRI) annual report on the Top 10 Health Technology Hazards, 2024 has identified “Usability challenges with medical devices in the home” as the number one hazard [8], noting that:

The number one topic addresses what has become a critical challenge—design shortcomings in home-use devices. When a device is developed, it's critical that human factors and the end user be considered to prevent misuse and harm. However, home-use devices are often not designed with the lay user in mind, and home users generally lack expertise in their use.

This motivates work which not only considers users from a more nuanced perspective than novice or expert, but also considers the impact of different dimensions of usability, in our case learnability.

Given the increasing prevalence of home-based care, the ability for users to rapidly and reliably learn how to operate medical devices has become a critical safety concern. Unlike traditional clinical environments, home contexts often involve first-time or occasional users who may be under stress, time pressure, or even facing an emergency. In such scenarios, usability alone is not sufficient, rather learnability (which we might initially consider as the speed and ease with which a user can acquire necessary skills) becomes a key determinant of both safe operation and effective intervention. By explicitly focusing on learnability, we address a gap in current usability evaluations which directly addresses the risks highlighted by ECRI and others. In addition, as we will discuss in Section 3, a clearer understanding of types of users and their experiences enables a view of both usability and learnability that may be better suited to the complexities of medical device usage in multiple contexts.

Furthermore, as discussed by Chaniaud et al. [5] learnability is typically not explicitly considered in usability assessments of home connected medical devices. In their work with an at home post-operative device they noted that “this rapid handling means that users need to know how to use the device from the very first attempt”, or rather that there is very little room for error. These high stakes environments emphasise the importance of learnability for medical devices and the need for specific tools to allow investigation of this key aspect of usability.

In order to analyse the learnability of a system, we must investigate learnability with regards to the intended target audience. Our above definition of learnability makes it clear that learnability is impacted by the end users' experience level, domain knowledge and pre-existing familiarity with a system. Within the medical domain, not only does learnability have the potential for significant negative consequences for its end users, but the end-users are a growing, diverse set of people. We must consider all users, from those with medical expertise, such as a nurse or doctor, to novice and first-time users and everything in between. While training may be used to assist in increasing learnability over time, when adverse events occur, the ability to use a system quickly to assist the person connected to the device is critical to ensure their safety and well-being.

As an example we consider continuous glucose monitors (CGM) used by people with diabetes. As of 2021, diabetes affects over half a billion people worldwide, making it a major health issue faced globally [33]. Controlling Blood Glucose (BG) levels is crucial in the management of the disease, with failure to control levels resulting in various health complications in different parts of the body, including the eyes, kidneys, feet and heart [26]. The advancement of diabetes technology, such as automated Insulin Pumps (IP) or CGM, means people with diabetes have more tools available to them with greater capability to help relieve some of the burden of managing their condition. However, when these tools fail, the consequences can cause serious harm or even be fatal [10], [18].

Documentation for IP and CGM often consider the primary or expert users of the system (i.e. the person with diabetes) and ensure that the system meets their needs, with appropriate training materials, thus making systems “learnable”. However, in reality, IP or other similar devices, are often used by secondary users such as parents (for their children) or teachers (for their students in classroom settings). In a medical emergency, such as a hypo- or hyperglycemic event, when BG levels become too low or high respectively, secondary users must be able to operate the device to avoid causing harm to those within their care. Therefore, it is important to investigate learnability of systems, not just in regards to the primary users, but also secondary users. In the event that the primary user cannot operate the device, it is likely that a secondary user will have to step in and perform vital tasks on their behalf.

The primary contribution of this paper is a methodology for the heuristic evaluation of learnability of medical devices for different types of users and use cases. First, we analysed an existing set of usability heuristics to identify which are applicable for learnability for different categories of users. From these results we developed a learnability matrix that incorporates the relevant heuristics for each category of user. In this paper we demonstrate the use of our heuristic matrix to evaluate the learnability of the Medtronic MiniMed 770G^1^ (MiniMed) focusing on two tasks that all key users may encounter. The rest of this paper is structured as follows: we begin with related work describing related usability and learnability evaluation techniques before defining categories of users. Next we introduce the learnability matrix and consider its applicability for the different user categories. We present a case study using the learnability heuristics for two specified tasks of the MiniMed IP. Lastly, we finish with a discussion on our findings finishing with concluding remarks.

## 2 Related work on usability and learnability

As described above, learnability is often considered as part of usability rather than separately. We therefore begin our consideration of related work with usability literature which considers approaches used in the domain of medical devices. We then focus on heuristic evaluation, which we rely on later for choosing appropriate heuristics to use in our approach. Following this we narrow our focus to learnability measures. Related work for user types and categories is considered later, in Section 3.

### 2.1 Usability, learnability and heuristic evaluation

To evaluate usability in software engineering contexts there are four primary techniques: formally, using an analysis technique; automatically, by a computerised procedure; empirically, by testing with users; and heuristically [25]. Within the medical domain, empirical research has been conducted into the usability of safety-critical devices such as diabetes technologies. For example, in [16] Kelly reports on surveys implemented with 158 type 1 diabetics (of which 84 were CGM users) which highlighted the high levels of distress experienced by end users resulting from poor usability and how this impacts psychosocial outcomes. Larsson et al. ran usability tests with both diabetic and non diabetic participants (10 and 20 respectively) and found that there were measurable differences in usability between similar diabetes technologies. Of the five pumps tested in their work, three were inaccurate for low dosages which would negatively impact small children if they relied on the device [19]. These highlight the importance of both usability testing in the safety-critical domain and also the significance of understanding how this can change depending on the profile of the user. In [35] Tu and Russo defined a visual checklist for evaluating the user interfaces of diabetes applications. They found that both qualitative and quantitative measures could be identified using this approach on the MiniMed Mobile application to consider its usability by people with type 1 diabetes.

In other domains, analysing usability also requires understanding user motivation. For example, Coyle and Peterson consider learnability evaluation for enterprise applications [6] and argue that business users typically have little to no choice about the application they use in the workplace which affects their motivations. To measure usability they looked at how well users retained information about how to use a system following first reading about, then interacting with a given software application. As such they aimed to measure not only initial usability without any instruction (walk up and use) but also retention of instructions and retention of use (after time and distraction). This is, of course, also relevant from the perspective of learnability. While motivation for use is different for users of diabetes technology, it is similarly true that they do not typically have a choice about the technology they can use (it usually depends on which devices are funded in the country they live in). This means that if the device they are using has poor usability they do not have the option to change to something else.

Heuristic evaluation has been used to evaluate safety-critical devices and is particularly important in the design and development process of such systems [17], [29]. Nielsen [23] reports that heuristic evaluation undertaken by 3 to 5 experts allows the discovery of over 75% of usability problems, making it comparable with empirical methods. Popular heuristic evaluation techniques include: the Ergonomic Criteria for the Evaluation of Human-Computer Interfaces [2], 10 Usability Heuristics for User Interface Design [24], and Eight Golden Rules of Interface Design [32]. However, as Lecaros et al. highlight, several heuristics were developed many years ago, and while their main principles remain relevant, they must be adapted to suit newer software platforms [20].

Several systematic literature reviews have been conducted on heuristic evaluation techniques. One such study by Fernández and Macías [9] reviewed a variety of heuristic sets developed since the 1980s and based on these also described ways of developing new heuristic sets. Jimenez et al. [15] focused instead on identifying which usability heuristics are being used most often in literature for evaluating software systems. Out of the 57 articles reviewed they identified the most popular heuristics as: Nielsen's 10 usability heuristics for user interface design [24] and Zhang et al.'s usability heuristics for patient safety of medical devices [39]. Further exploration of Zhang et al.'s approach shows that their heuristics were developed using Nielsen's heuristics and Schneidermann's eight golden rules of interface design [32] as their basis. As such, the authors refer to their heuristics as the Nielsen-Schneidermann heuristics. In [7] de Queiroz Pierre explores the differences and similarities in heuristic frameworks, investigating 33 sets of frameworks from 1986 to 2012. A key result of this work was that they found multiple different heuristics served the same purpose, with similar principles outlined in each for defining usability.

### 2.2 Learnability

To measure learnability, Schmettow et al. [31] propose a special protocol, including multiple performance measures and repeated sessions to the training process. They argue that one of the problems with existing usability validation testing of medical devices is a prevailing focus on cross-sectional validation studies, ignoring the issues of learnability and training. This highlights the challenges and stress involved for end users to learn how to use medical devices like IP and the need for adequate processes for investigating learnability of these systems. In both Schmettow et al.'s work and similar work by Chaniaud et al. [5] there is an understanding that users ‘learn as they go’, i.e. that learnability becomes easier over time. In contrast, our focus is wider, in that we consider both adverse events (which requires novice users to ‘walk up and use’ systems) as well as different categories of typical users.

A focus on measuring learnability over time is also seen in the works of Santos and Badre [30]. Their ‘discount learnability’ uses the concept of cognitive units, or ‘chunks’ with the hypothesis that as users gain experience (and learn) the cognitive chunks get bigger. This has potential to be used as a measure of learnability over time (by graphing the cognitive chunks).

In work on learnability for novice users, Linja-aho defines learnability as “how quickly and comfortably a new user can begin efficient and error-free interaction with the system, particularly when he or she is starting to use the system” [21]. The goal of learnability is then considered to be “efficient and error-free” interaction. Linja-aho's focus on novice users resonates with our work where we consider novices as a crucial category of users (discussed in more detail in Section 3). The work identifies eight learnability factors related to the user interface: Visibility of operations; Feedback; Continuity of task sequences; Design conventions; Information presentation; User assistance; and Error prevention. These are then further divided into 28 learnability guidelines. Additional factors related to user expectations and training are also provided, but these are less relevant for the work we focus on.

Other studies have also investigated training and its impact on learnability for medical systems, including the difficulties users may face [22], [28]. Medication errors continue to occur with infusion pump devices in hospital settings with rigorous reporting and training processes required to address use errors [34]. Hernandez discovered patients' knowledge of diabetes management systems varies from person to person, and therefore the training they receive needs to be personalised in order to be effective [12]. Grossman et al. [11] highlight learnability as one of the most important aspects of usability and also consider user types. In their survey of learnability definitions they explore how learnability is traditionally defined in relation to the skill levels of primary users (e.g. novice, typical, new, experienced). However, many of the studies discussed here focus on “users” as one set of people and do not differentiate between the needs of various groups, generally referring to the primary user group only. We argue that considering learnability of a system from different users perspectives is important to ensure well-designed systems that do not negatively impact the health of end users and adequately meets their needs (discussed further in Section 3 below).

In this paper we take the approach of modifying and adapting existing heuristic sets (in the manner of Zhang et al. [39] and using Zhang's heuristic set as the basis) rather than basing heuristics on empirical data. This allows us to consider the nuances of different categories of users without having to rely on real world data, which in a medical context can be difficult to obtain. We build on the approach of Grossman et al. [11] by developing a matrix which considers learnability for categories of users that can provide an initial understanding of the learnability of a medical device without the need to conduct a user study. This is particularly important in the medical domain where we do not want to subject users to adverse events, even in an artificial setting, simply to perform initial evaluation of the learnability of a device. Of course this does not mean that usability studies with real users should not be performed, once a device can be shown to have reached a level of maturity where it is believed to be ready for release then such studies are essential.

## 3 Categories of users

The importance of understanding users is a central concept within the field of Human Computer Interaction (HCI). While there are many different ways of achieving this (which can be loosely grouped under the heading of ‘user-centred design’ (UCD)) the goal is to ensure that the systems and interfaces we design are usable (see definition in Section 1) by the intended users. In some cases the intended users are a very specific group who may be defined by specific tasks. For example, if we consider aircraft cockpit software, it will only be used by a very specific group of users (pilots) for very specific tasks (controlling an aircraft). We can also assume that all of these users will have had extensive training in using the software as part of their flight training. In contrast, something like a web browser could potentially be used by any person with any level of skill and experience, from a first time user (novice) to an expert. In this case the category of tasks being performed may also be diverse and so we cannot make assumptions about the users' skill levels for any given task. For specific use cases for a piece of medical equipment, where the tasks are well defined but the users may be diverse (such as the one we consider in this paper) it is useful to consider ways of categorising users, based on their experience with the device and purpose of use. This then allows us to consider their specific needs or use cases as they relate to both usability and learnability.

The International Organisation for Standardisation (ISO) standard ISO 9241-210:2019(en), defines “user groups” as a: “subset of intended users who are differentiated from other intended users by characteristics of the users, tasks or environments that could influence usability”. Our primary focus is on the first of these, the characteristics of users. In order to ensure learnability for all possible users of a medical device (specifically the Medtronic MiniMed in this paper) we need to understand which of these characteristics impact learnability. Literature which seeks to consider the diversity of users groups typically focuses on demographic aspects (age, gender, educational, socioeconomic background), domain and technical aspects (profession, role, expertise), aspects of use (interests, motivation, intent and task) or a combination of these. Central to such work is the ability to identify users prior to development in order to properly consider them throughout the design and development process. For ‘walk up and use’ problems (where the user could reasonably be anyone) broader categories such as “general public” or “expert vs novice” may be used, but this can be problematic. In work examining the categorisation of users for digital cultural heritage systems, Walsh et al. argue abstract and generic groups can mask specific sub-group needs and often a more nuanced view is required [38]. They also noted that in the work they surveyed the broad classes of expert/professional, semi-expert/hobbyist and novice/non-expert emerged as common distinctions. The dimensions against which users are categorised into these groups broadly fall into domain knowledge, technical expertise and motivation.

In [14], Janhager builds on Buur and Windum's classification [4] of primary and secondary users with the addition of ‘side-users’ and ‘co-users’. Respectively, these are people who are affected by a product's use or those who use the product but not for its' intended purpose. Similarly, the types of users we need to consider also encompass more than just primary or secondary users, but are also different from Janhager's definitions. We describe instead “Passive Users” and “Ad Hoc Users”, as defined in Table 1 below.

**Table 1:** Categories of Users for the MiniMed 770G.

User	Definition	Training	Expertise
Primary	Uses the device for themselves	Yes	High
Secondary	Carer for someone needing the device	Yes	High
Passive	Patient who needs the device but does not control it themselves	No	Medium
Infrequent	Occasional carer for someone needing the device	Yes	Low
Ad Hoc	Unknown person who interacts with the device in an emergency	No	None

**Fig. 1:**
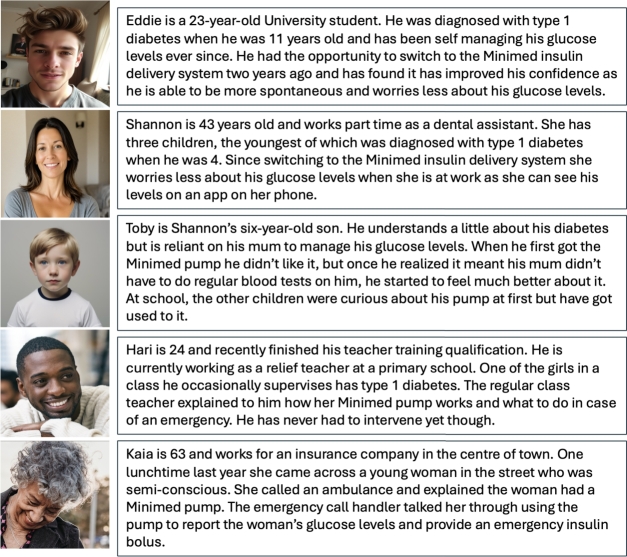
Personas for User Archetypes. Images created by authors using AI image generator https://www.imagine.art/.

We elaborate on these types of users further and then exemplify them as archetypal personas. These are not intended as fully-fleshed personas that can be relied on as representations of actual users throughout the design process, the challenges of which have been identified and documented in [37]. Rather they act as representations of specific learnability attributes we wish to consider and by personifying these archetypes we have a single reference point for each. The primary user can be considered an independent user of the medical device. They have been trained in its use and have autonomy in controlling its use, as exemplified by the persona of “Eddie” in Fig. 1 (Personas for User Archetypes). The secondary user is a carer of the person who needs the medical device (“Shannon” in Fig. 1). This category might also include medical personnel who interact with primary users as patients. The passive user is a dependent person who relies on the device, but is unable to control it themselves (“Toby” in Fig. 1). The infrequent user will occasionally be called upon to support a person who needs the medical device (“Hari” in Fig. 1), they will have some training but may use the device very infrequently. Finally, the ad hoc user may be a bystander or passer-by who encounters a person using the device who is temporarily unable to control it themselves (“Kaia” in Fig. 1).

The level of expertise these different users have is based on several factors: training, regularity of use, and familiarity with device. The highest level of expertise is seen in the users who have been trained and who use the device regularly. Personas like Toby do not use the device themselves, but are very familiar with use as they see their carer using the device for them on a regular basis. This might make them more of an expert than the casual user, whose training may have been a significant time period prior to actually using the device. Finally, the ad hoc user cannot be expected to have any familiarity with the device or even know its purpose.

While we have described personas to fit our specific group of users, these are not fixed. Both time and use over time can move a user along the dimension of experience. Time may also shift users from one type to another (for example, a passive user grows up and becomes an independent primary user). There are also other factors that may impact the user type. A primary user experiencing a medical event may be incapable of controlling the device themselves and therefore become a passive user. In addition, there are other attributes which may affect the knowledge of all users which relate to their understanding and interest in technology. A study of home haemodialysis patients performed in 2015 identified different coping styles adopted by patients managing their own health conditions and technology [3]. These included those who proactively sought to understand everything about the device in use (in this case home dialysis machines) including troubleshooting in order to feel fully in control. Conversely, there were also users who preferred to relinquish control wherever possible, to both medical staff and family members assisting with care.

## 4 Learnability matrix

As discussed above, we define *learnability* as the ability for a user to learn how to use a system depending on their experience level, domain knowledge and familiarity with a system. Additionally, when considering medical devices, carrying out a user study to determine learnability is neither feasible nor ethical, as it may cause unnecessary distress to the participants. Therefore, we use heuristic evaluation as a suitable alternative to define the learnability matrix. This is particularly beneficial as it allows us to consider the different categories of users as well as allow designers to evaluate a system without the need to involve users directly in a user study. This evaluation may be repeated throughout an iterative development process. Note that this is not a replacement for typical usability analysis but should instead complement existing best practices in the medical domain.

While there are several usability heuristics, there are none, to our knowledge, that investigate learnability specifically. As described in Sections 1 and 2 learnability is considered a key aspect of usability, which means that learnability is investigated in existing heuristics, but indirectly. Therefore, our approach uses an existing heuristic set for medical devices defined by Zhang et al. [39] to create a matrix which will allow us to evaluate learnability (see Table 2).

**Table 2:** Zhang's Usability Heuristics for Medical Devices [39].

Name	Definition
Consistency & standards	*Users should not have to wonder whether different words, situations, or actions mean the same thing. Standards and conventions in product design should be followed.*
Visibility of system state	*Users should be informed about what is going on with the system through appropriate feedback and display of information.*
Match between the system and world	*The image of the system perceived by users should match the model the users have about the system.*
Minimalist	*Any extraneous information is a distraction and a slow-down.*
Minimize memory load	*Users should not be required to memorize a lot of information to carry out tasks. Memory load reduces users' capacity to carry out the main tasks.*
Informative feedback	*Users should be given prompt and informative feedback about their actions.*
Flexibility & efficiency	*Users always learn and users are always different. Give users the flexibility of creating customization and shortcuts to accelerate their performance.*
Good error messages	*The messages should be informative enough such that users can understand the nature of errors, learn from errors, and recover from errors.*
Prevent errors	*It is always better to design interfaces that prevent errors from happening in the first place.*
Clear closure	*Every task has a beginning and an end. Users should be clearly notified about the completion of a task.*
Reversible actions	*Users should be allowed to recover from errors. Reversible actions also encourage exploratory learning.*
Use users' language	*The language should be always presented in a form understandable by the intended users.*
Users in control	*Do not give users the impression that they are controlled by the systems.*
Help and documentation	*Always provide help when needed.*

**Fig. 2:**
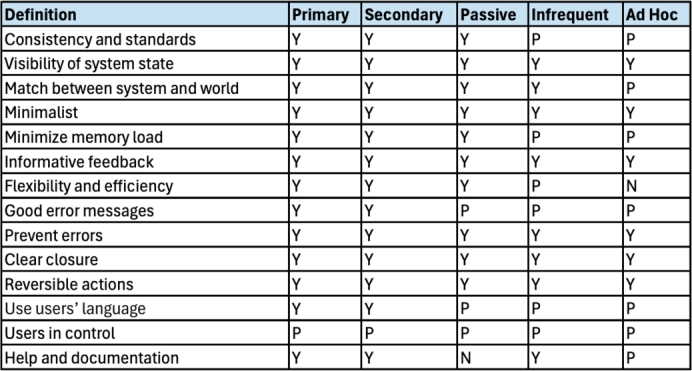
Learnability Heuristics for User Categories.

For each heuristic in Table 2, we compared the individual heuristic against each category of user as depicted in Fig. 2. For these heuristics we were interested in how learnability was considered within each definition and how this changed depending on the users' context of use with a medical device. For each heuristic we determined learnability to be considered (“Y”), partially considered (“P”) or not considered (“N”). The heuristics were reviewed individually before comparing and contrasting results to finalise the table.

As learnability is a key aspect of usability, it was evident that each of the heuristics would have some element of learnability embedded in its definition. In Fig. 2 we can see that when considering learnability there is no difference between the primary and secondary user. However, while these two users are very similar, they are not identical, particularly as a primary user may become a passive user during an adverse medical event. Also, as described above, the work by [3] shows that primary users have different relationships with their devices which may lead them to be less engaged with learning their use beyond the bare minimum. All heuristics fully considered learnability from the primary and secondary users' perspective with the exception of Users in Control. This heuristic was partially considered when we focus on learnability as it relates to avoiding surprise rather than users learning how to complete a task.

Given heuristic sets are often developed with a primary user in mind, secondary and primary users being similar is not surprising as both will interact with a device to care for themselves or others. The only difference between the two types of users is who is connected to the medical device and how often they interact with the device. However, when we start to consider the passive, infrequent or adhoc users, both learnability and the heuristic set changes.

Passive users are different from primary or secondary users in two ways. The first is in relation the users' language. If we consider the persona of Toby in Fig. 1 (Personas for User Archetypes), he may be connected to a pump but not familiar with the medical language the device uses. In this case applying this heuristic from the point of view of Toby is only partially considered, as the device's language would need to be changed for Toby to understand and comprehend. Similarly, help and documentation for Toby is not considered by the heuristic set, as the manuals and other documentation associated require a higher level of reading comprehension.

For infrequent users the majority of heuristics are either considered or partially considered. Again, consider the persona of Hari in Fig. 1 (Personas for User Archetypes), in terms of something like “Match between the system and the real world” it will be important for him to be able to recognise common icons or actions based on interactions with similar devices and build on his existing mental model in order to use the device. Conversely, “Flexibility & efficiency” is only partially considered in Hari's context, as it is unlikely he will be able to remember different ways of interacting with the device other than the way he was originally taught because he uses it infrequently. Similarly, for other heuristics in the set, the amount of time an infrequent user can be expected to interact with a medical device impacts the level of learnability we can investigate from the heuristic set.

Lastly, our adhoc user category, has similar considerations to our infrequent user but these are impacted even further by the minimal amount of time they may have used a device. They are essentially the ‘walk up and use’ end user who may never have interacted with the device before. In terms of the heuristic set, items like minimalist design are key to make it easier for them to find the options they are looking for and reduce memory load and burden. However, in contrast to the infrequent user, flexibility and efficiency of use are not considered as part of the heuristic for learnability, as the adhoc user has no prior mental model to know about short cuts and different ways to complete tasks. Furthermore, the use of help and documentation is only partially considered here as it is unlikely that the user will have access to help and documentation outside of what is available on the device.

It is evident from considering the heuristic set from the perspective of our different user categories that the way in which we can analyse learnability of a medical device changes depending on the user. This is because our user categories allow us to consider different perspectives which in the context of medical settings is crucial to ensure devices can be operated safely by different types of users with various skills, training and ability. Next we introduce our case study device, the Medtronic MiniMed 770G system utilising two commonly used tasks to highlight how heuristic analysis changes for each different user category.

## 5 Case study: medtronic MiniMed 770G system

**Fig. 3:**
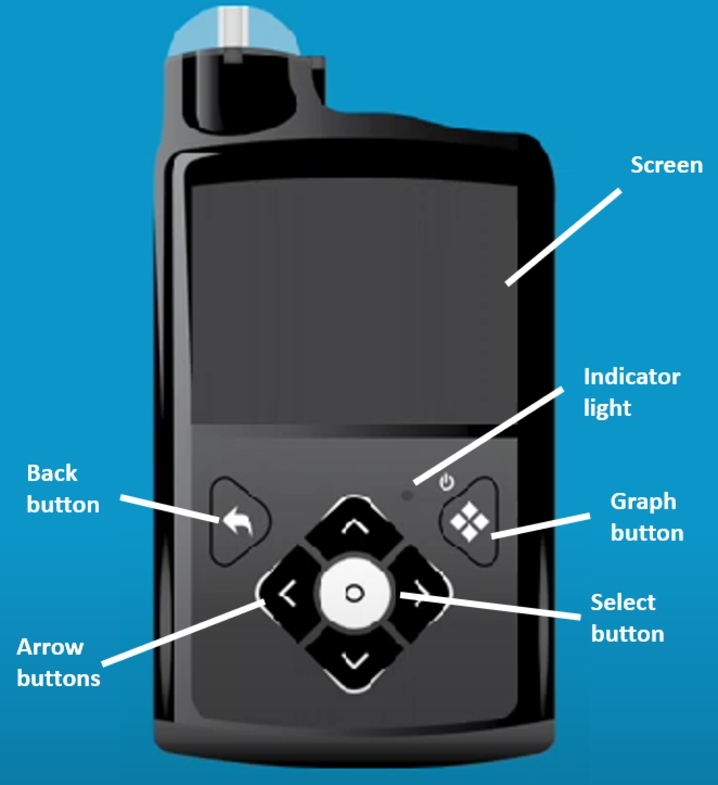
The Medtronic MiniMed 770G System.

The MiniMed IP system is a device that delivers insulin subcutaneously (see Fig. 3). It is designed to mimic a human pancreas, continuously infusing small amounts of insulin to manage the amount of glucose present in the bloodstream. The MiniMed achieves this by delivering insulin from the pump to an infusion set attached to the body (usually the abdomen, outside of the thigh or back of the upper arm). The infusion set consists of a small tube inserted under the skin (called a cannula) and an adhesive patch to keep the set in place. The pump is similar in size to a smartphone and users typically store the pump in a clothing pocket, bag or attach it to their waistband.

The device has two main modes the users can choose from: automatic and manual. The automatic mode, SmartGuard, offers Hybrid-Closed Loop (HCL) functionality and can only be used if the pump is paired with a CGM, allowing the device to work as an Artificial Pancreas (AP) system. Manual mode, as the name suggests, requires input from the user to initiate actions instead of allowing the device to run automatically. There are several variants of manual mode which support the use of a CGM in conjunction with the pump if the user wants to take advantage of continuous glucose monitoring while retaining full control of the system.

To investigate the learnability of the device for this case study we used a digital prototype provided by the manufacturer^2^ and the official user manuals.^3^ As our interest was in safety-critical contexts, for the first task we focused on the ability to suspend and resume insulin delivery as may occur during hyper- or hypo-glycemic events. For task two, we investigated updating the basal pattern, a task which should only be completed by an advanced user of the system.

### 5.1 Suspending insulin delivery

To suspend insulin delivery no BG level changes should have occurred within the last 12 minutes and there must be no active bolus insulin. If changes have occurred in the last 12 minutes the system will prevent insulin suspension and notify the end user. The process to suspend all insulin delivery involves unlocking the device, opening the main menu and selecting the ‘Suspend Delivery’ option which is located near the bottom of the screen. The user must confirm their choice which triggers a new screen stating ‘Delivery Successfully Suspended’. A similar banner will also appear on the home screen. Note that when all delivery is suspended, the functionality of the device becomes limited as critical features are disabled.

### 5.2 Resuming insulin delivery

To resume insulin delivery we must assume that the delivery has been paused, which is communicated via the banner on the home screen described above. The process is similar to suspending delivery in that the user must unlock the device and open the main menu. Near the bottom of the screen they must locate the ‘Resume Delivery’ menu item. Again, a confirmation message will appear and the device will notify the user that the delivery has been successfully resumed. On the home screen the “Suspended Delivery” banner is no longer present.

### 5.3 Updating the basal pattern

In this task a user wishes to update the basal pattern for the device. We assume the pump is locked, there is only one basal pattern, and no BG level has been entered in the last 12 minutes. Furthermore, there is no active insulin other than basal, i.e. there is no bolus. The user must unlock the device and navigate to the Basal menu. Once inside the menu they are presented with the current basal rate, from here they can select the ‘Delivery Setup’ item and ‘Basal Pattern Setup’ option, the user may then edit the basal rate and timings. Once changes are confirmed the new Basal rate is saved.

### 5.4 User categories

Each of these tasks are relevant for each of our user categories. We demonstrate this by using our personas from Fig. 1 (Personas for User Archetypes) and scenarios in which they may complete the task. Firstly, as primary and secondary users, both Eddie and Shannon may wish to suspend and resume an insulin delivery for several different reasons. For example, devices often must be removed when showering or swimming as they are not waterproof. It is typical to suspend delivery and remove the device before reattaching the device afterwards and resuming delivery. In this scenario neither Eddie or Shannon are likely to be in a safety-critical situation as they will simply resume delivery when the activity is complete. Additionally, Eddie and Shannon will also be familiar with the meaning behind updating a Basal pattern, they will both have understanding of the context around changing a rate and be able to do so accordingly. However, Eddie will be able to make a more informed decision based on how he is feeling, whereas Shannon must make a decision based on her intuition on Toby's typical insulin use and relying on him relaying this information to her.

Next we consider Toby, as the passive user (with Shannon being the secondary user) he does not directly interact with the device. Therefore, from his perspective his parent simply starts and stops insulin delivery so he can get on with a water-based activity. Again, this is unlikely to be a safety critical situation or one in which Toby learns anything more about the device than it cannot get wet. Similarly, he may not have full understanding around the questions that his Mum is asking regarding updating his Basal rate, it is unlikely that he is able to clearly articulate how he is feeling, leaving Shannon to rely on the data provided by the pump or CGM to make an informed decision.

Suspending and resuming insulin delivery for both infrequent or adhoc users are likely to occur during adverse events in order to trigger the user to interact with the device. From Hari's perspective, he would only interact with the device to prevent or help during a hypo- or hyper-glycemic event. Similarly for Kaia, as she does not regularly interact with the device, she would only encounter the device in an emergency. Therefore it is key that she is able to be talked through both of these tasks, in order to help the primary user.

However, when we consider updating the basal rate these two categories of user differ. Hari as an infrequent user may be asked by a parent to check the basal rate is correct but unlike a primary or secondary user he is unlikely to need to change this unless directed by a caregiver like Shannon to do so. In contrast, Kaia would not be expected to interact with the Basal rate as this would not provide the immediate assistance required in an emergency event. It is unlikely that Kaia would investigate the Basal rate further in an adverse event other than what is displayed on the home screen.

## 6 Results

For each task the heuristic set was evaluated to determine adherences and violations. This was followed by a secondary analysis where each item was considered from the perspective of each user taking into consideration if learnability was relevant for this heuristic item from the matrix in Fig. 2. The results of each task are described next.

### 6.1 Suspending insulin delivery

**Fig. 4:**
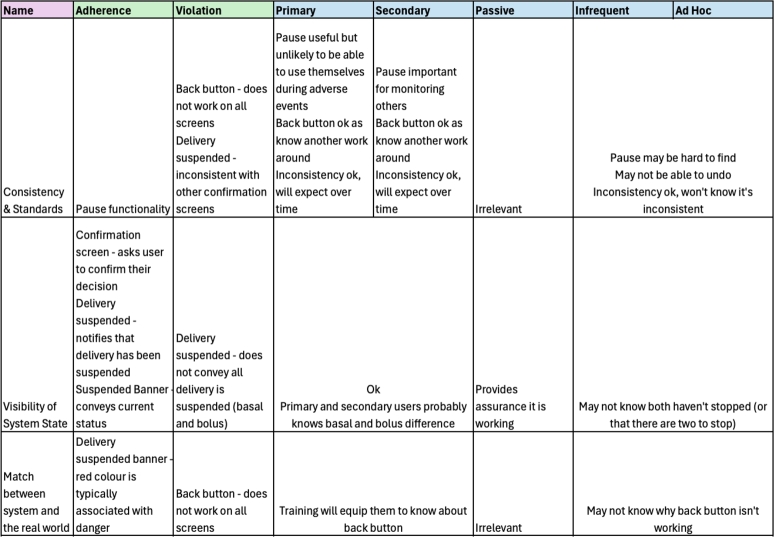
Evaluation for Suspending Insulin Delivery with Violations for each Category of User.

**Fig. 5:**
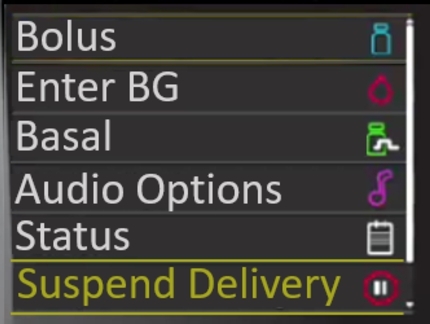
Suspend Delivery is Hidden and Requires Navigation.

There were 18 adherences and 9 violations identified during the heuristic analysis of this task (see supplementary materials). A summary of these is given in Fig. 4. The suspend delivery option essentially operates as a “pause” for the device by ceasing delivery of all insulin, this is important as it allows users to immediately stop delivery of insulin during an adverse event or, as discussed above, prior to removing the device for activities like swimming, showering and so on. This functionality is consistent with other medical devices to ensure a device can be turned off immediately if necessary. However, when we consider the different perspectives of the user categories, this changes the way we interpret the heuristic analysis. In the case of a primary user, they would not use the suspend delivery function themselves during an adverse event if they were incapacitated and they would rely on another user (secondary, infrequent or adhoc) to interact with the device for them. This highlights the importance of these other users being able to locate the functionality in a timely manner. A secondary user will have had training with the device and is therefore more than capable of completing the task successfully, while the infrequent and adhoc user may struggle to locate the menu item hidden in the device menu without further navigation or guidance (see Fig. 5).

Our analysis showed that the system allows users to complete the task in more than one way, offering flexibility to the end user. For primary and secondary users this allows them to use shortcuts and build a mental model to understand the different paths through the system. In contrast, for passive users these options are irrelevant as they are not directly using the device. A theme common throughout the majority of the analysis in this particular scenario as we consider the persona Toby who does not actually interact with the device himself. Note that for this scenario however, visibility of system state is important for a passive user like Toby, as it provides assurance that the device is working. For infrequent users like Hari, flexibility is not as important as knowing one way of achieving a task is enough, reversal of actions using the back button however will help him to recover from mistakes easily.

A key violation for this task was in relation to the back button as it did not work on all screens, primarily when prompts are available. In the case of primary and secondary users, training will equip them to know when the back button is usable and when it is not, while for infrequent or adhoc users they may not understand why the back button is not working or that it is simply a standard behaviour of the system. This lack of control could potentially cause panic or discomfort for these users, particularly if using the device during an emergency.

The use of a confirmation screen allowed users to confirm their actions providing: clear visibility of the system state; informative feedback and ensuring the user is in control; preventing errors, if they do not want to suspend they can simply cancel; and clear closure of the task. For all users, regardless of their category this confirmation allows them to confirm the task is complete and helps the system communicate that they have finished successfully. This is a clear example of when the user categories do not impact the heuristic evaluation.

**Fig. 6:**
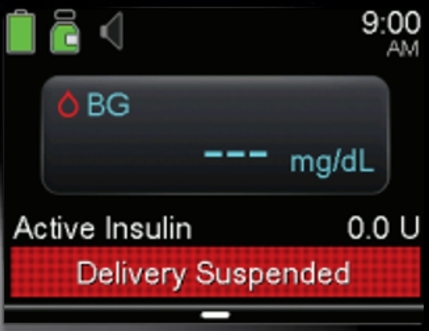
Basal and Bolus Delivery Suspension not Identified.

There are two types of insulin delivery that the MiniMed is capable of, basal and bolus delivery. Basal delivery occurs continuously throughout the day in small amounts while bolus is used to manually correct insulin levels, such as when they may spike due to food intake. When suspending a delivery is completed both the basal and bolus delivery is paused. The “Delivery Suspended” banner, as shown in Fig. 6, clearly shows the system status to the end user and notifies them that the task has been successful. However, while primary and secondary users will know the difference between basal and bolus, and know the implications of both being stopped, infrequent or adhoc users may not understand the impact this could have if paused for a long period of time. A simple change to this message “All Delivery Suspended” would notify users that both bolus and basal have been fully disabled. While an infrequent or adhoc user may not know what this means it would at least notify them two types of delivery have been paused. Furthermore, the use of the colour red signifies that a task has stopped. However, red can also be used to convey an emergency, for an adhoc user this may cause panic and concern.

### 6.2 Resuming insulin delivery

**Fig. 7:**
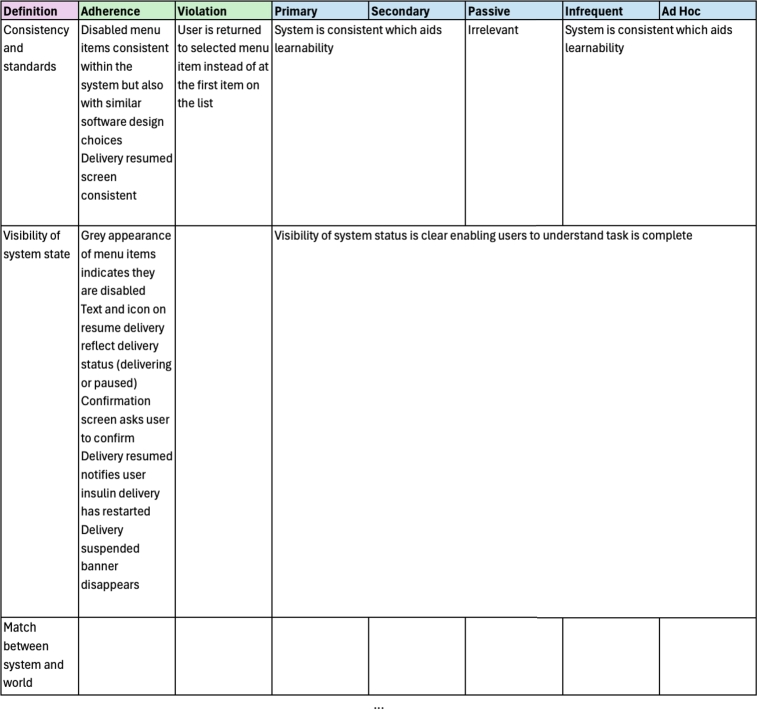
Evaluation for Resuming Insulin Delivery with Violations for each Category of User.

There were 20 adherences and 6 violations identified during the heuristic analysis of this task (see supplementary materials). A summary of these results is given in Fig. 7. Note that the results for heuristic three for this task are blank, this demonstrates that there were no adherences or violations identified for this heuristic, removing the need for further learnability analysis for each user category.

**Fig. 8:**
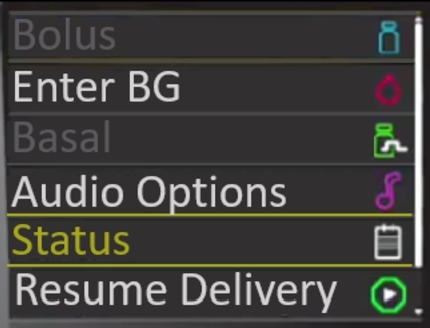
Disabled Menu Items.

For the system to resume insulin delivery it must currently be in a paused state. This is indicated to the user through the use of the “Delivery Suspended” banner, as discussed in the previous task and shown in Fig. 6. To resume the delivery the user must open the menu, as shown in Fig. 8. The disabled menu items are consistent within the system but also with other interface design choices, this consistency aids with learnability for all user categories with the exception of the passive user as they do not directly interact with the device itself. This ensures that the visibility of the system state is clear and that both bolus and basal cannot be modified until the delivery is resumed. Furthermore, this helps to prevent errors as options that are currently unavailable are disabled in the menu. The change of icons on the menu item and text from “Suspend Delivery” to “Resume Delivery” further reinforces that the delivery is currently suspended.

When the menu is opened again the system will remember the last selected option, in this case “Resume Delivery”. This is not the same behaviour as when the menu is opened to suspend delivery. While this aids in speeding up the interactions for a primary or secondary user, infrequent and adhoc users may not be expecting this behaviour. In addition, this makes finding “Resume Delivery” much easier than finding the option for “Suspend Delivery”. With regards to our earlier result for the suspend delivery functionality, it would have been easier to locate if this was the first item on the menu, that is, permanently relocating the item to the first option in the menu. This would have a positive impact on the learnability of the device for infrequent and adhoc users as they are less likely to use other options. In addition, as this has no impact on primary or secondary users this change can be made without negatively impacting on the user experience.

**Fig. 9:**
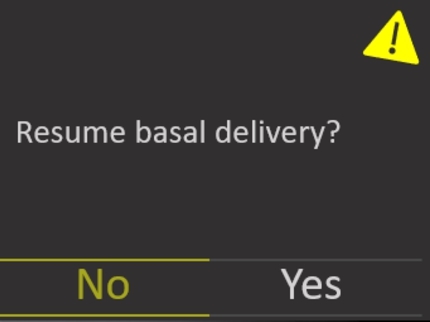
Resume Delivery Confirmation.

When the user asks the system to resume the delivery they are presented with the resume delivery screen (Fig. 9). Note that this explicitly states that basal delivery will be resumed and not bolus. Similar to our discussion above for the “Delivery Suspended” banner, while primary and secondary users will understand from training that they need to restart a bolus (if still necessary) an infrequent or adhoc user may not understand the difference or the impact this could have on the person connected to the pump. This may make them concerned that the task is not complete and lead them to explore the bolus options further.

**Fig. 10:**
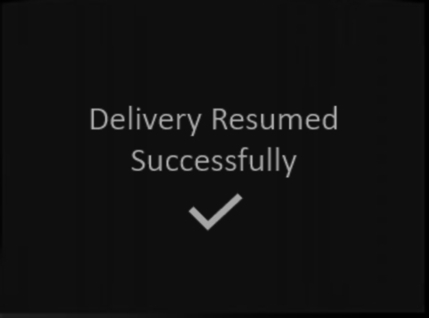
Delivery Resumed Successfully.

In addition, when the task is complete the system states that “Delivery Resumed Successfully” (Fig. 10). While this clearly communicates the system status to the user with informative feedback it may further confuse the user as to whether both basal and bolus have been resumed. For primary and secondary users, assuming experience and training with the device, they are likely to understand simply that the task is complete. In contrast, for infrequent and adhoc users, they may not understand what the message means or what further impacts this may have. However, for all users this provides a clear closure, in that they can trust the task is complete. This message is also consistent with our previous task, which further aids learnability of the system.

### 6.3 Updating the basal pattern

**Fig. 11:**
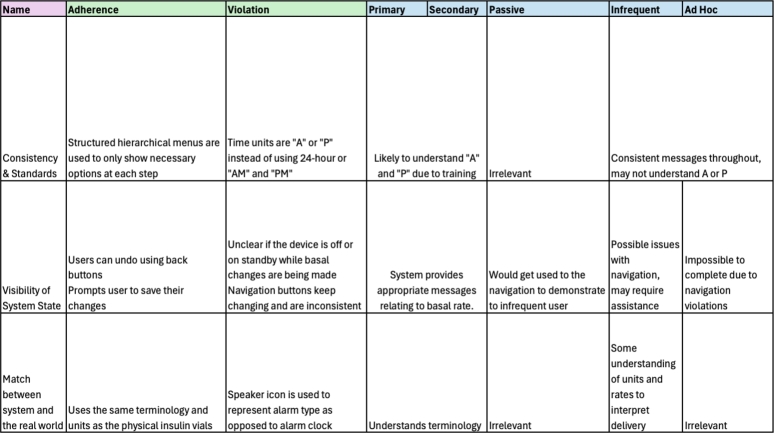
Evaluation for Updating the Basal Pattern with Violations for each Category of User.

There were 19 adherences and 12 violations identified during the heuristic analysis of this task (see supplementary materials). A summary of these results is given in Fig. 11. In this task we see some significant differences between each category of user as a result of the heuristic evaluation and learnability focus of our secondary analysis.

**Fig. 12:**
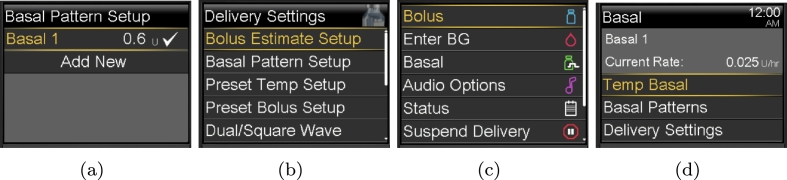
Sub-menus for Updating the Basal Rate.

In order to modify the basal pattern the user must navigate through several structured hierarchical menus to edit the basal rate (see Fig. 12). While necessary options are only shown at each step, it places burden on the user to remember which step in the process they are up to. Once inside the ‘Basal Patterns’ menu they are then presented with the current rates and the option to add a new rate or edit an existing one. This represents both an adherence and violation in the heuristic evaluation, as it adheres to a minimalist design, crucial for user interfaces with such small screen real estate, while violating minimising memory load as the user must keep track of what step in the process they are currently up to. From a learnability perspective, it may take several interactions with these menus to remember and understand how to edit the basal rate successfully.

However, if we consider each of our user categories, for primary and secondary users it is likely that can follow this structure due to the training and resources provided for the device. For a passive user this is irrelevant as they would not be updating values directly themselves. For an infrequent user they can follow the wizard-like interaction, however, no support or extra detail is provided meaning they may end up lost in the menu hierarchy unable to find the right setting. Similarly, for an adhoc user this interaction is likely impossible due to lack of appropriate help and documentation on the device.

These learnability issues are further compounded by the difficulty of navigation in the task. The button used to return to the previous screen keeps changing throughout each step and it is unclear which button to press to return or undo an action, making it difficult to learn how to complete the task. A primary or secondary user who may complete the task repeatedly would likely learn and remember which button to press to return or undo their actions. Similarly, when we consider a passive user like Toby they may pick up on this navigation by watching a parent like Shannon complete a task, which they could use to demonstrate the behaviour to an infrequent user.

In contrast, an infrequent user like Hari is unlikely to be able to navigate the task without some assistance as the interactions are not obvious due to the changing back buttons, especially when coupled with some of the domain specific language used. For instance in the Fig. 12 (d) an adhoc user may assume “Basal Patterns” would allow them to edit the basal settings but this displays the current patterns, they must navigate to the “Delivery Settings” option instead. As a result of this navigation, an ad hoc user is likely to find this task impossible, instead getting lost in the hierarchical menus and unable to undo their actions.

**Fig. 13:**
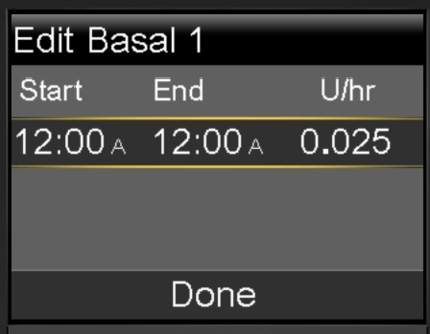
Updating a Basal Rate.

In Fig. 13 we can see the edit screen for the basal rate. This represents several violations for the heuristic evaluation as the “Start” time field cannot be updated, despite looking the same as the other fields. In addition, “A” and “P” are used to represent “AM” and “PM” respectively to save space on the screen which is inconsistent with other screens (see Fig. 6 above). For primary or secondary users they have likely gained understanding of the terminology due to their frequent experience with the device and familiarity with diabetes. Similarly, for a passive user, while it may not speak their language, they may gain some understanding through watching a secondary user interact with the device.

Our perspective on learnability of the device changes however when we consider the infrequent user. The infrequent user like Hari may not understand why a start field cannot be changed, making them unsure how to complete the task. Furthermore, the device does not use language they are familiar with, they may confuse the “A” or “P” as medical units as opposed to times. Meanwhile for an adhoc user like Kaia, these considerations are irrelevant as they are unlikely to be able to reach this screen which is more than seven menus deep into the hierarchy of the device.

## 7 Discussion

The learnability matrix in combination with the user categories provides a structured way to assess medical device learnability from different user perspectives as highlighted by our case study in Section 6. By performing this secondary analysis considering the impacts for each user category we are able to determine gaps that a standard heuristic evaluation may overlook. Pairing each of the these categories with specific personas further emphasises the need to consider the different user perspectives and assists in bringing each of the categories “to life”. Rather than asking what may impact a passive user we can consider how Toby may be directly affected, in terms of learnability, by the design choices of the software engineers.

As stated previously, heuristic evaluation typically focuses on usability, where learnability is only one aspect of the evaluation [27]. While it can be hard to separate learnability from a wider usability analysis, using the existing heuristics in combination with the user categories as outlined in the matrix enables us to consider learnability specifically rather than just usability alone. Focusing on the user perspective allows us to consider key aspects of learnability, such as learnability over time, prior training, and familiarity with the domain or other similar devices [31], [5]. This secondary analysis changes the way we interpret the heuristics and consequently discover not only the issues inherent in the system, but also how they will impact each different type of user.

Furthermore, this analysis highlights the difficulty of separating learnability from usability given the large overlap between each definition. Even as the authors were performing their analysis they would often conflate learnability with usability and several iterations through the matrix helped to narrow the focus of the analysis to adherences and violations for learnability rather than usability. The personas were useful in this regard as they allowed us to focus on the impact of learnability with respect to the persona's level of expertise, reinforcing the importance of training and familiarity over time with the device and the direct impacts that this has on learnability.

With respect to medical devices specifically, the results above demonstrate that passive, infrequent and adhoc users are the most likely to be impacted by learnability issues, especially during emergency scenarios. As the use of these devices becomes more widespread it will be important for devices like the MiniMed to enable emergency response systems that guide users to assist the passive user (the person experiencing the adverse event in this scenario) to assist and enable the device to be learnable in a ‘walk-up-and-use’ situation. From the personas shown in Fig. 1 (Personas for User Archetypes), Kaia is only able to use the device because the emergency call handler talks her through the steps. However, given the diverse and wide range of medical technology available it is not reasonable to expect emergency handlers to have this knowledge for all devices, rather they may give more generic advice, such as “find the suspend delivery option to turn off insulin delivery” and rely on the user to achieve this. We argue that it is the responsibility of the engineers to not only consider these types of scenarios through the use of tools like the learnability matrix above, but also to identify implications and potential pitfalls to design systems capable of performing well in these scenarios.

In addition, by using the learnability matrix we were able to identify design improvements based on the findings, such as changes to message phrasing or movement of menu items. In particular, considering the level of domain knowledge a primary or secondary user may have when compared with an adhoc or infrequent user around topics like basal or bolus changed our analysis of the heuristics and consequently learnability. These changes in domain knowledge are critical for medical devices, especially when we consider safety and health impacts another user's decision may have on the passive user's health. Combining these techniques with rigorous formal testing and typical usability analysis is key to ensure safe and reliable systems.

As discussed in Sections 1 and 2, direct usability testing of safety-critical medical devices during real or mock adverse events poses serious ethical concerns, as it will subject users to unnecessary stress and harm. This heuristic-based approach provides a non-invasive way to analyse learnability without exposing users to risk. In this paper we assume taking this approach will result in picking up approximately 75% of errors as is the case for most heuristic analyses [23]. However, a limitation of this study is that this analysis was carried out by two primary researchers and it requires further validation to confirm this. In future work it would be useful to see how the matrix performs with other usability experts and how many learnability errors can be detected.

In this paper we have used a MiniMed IP as a case study to demonstrate the applicability of our approach to medical devices. However, this approach is applicable to other at home medical devices including those primarily used for data collection rather than medical intervention. For example, if we consider devices like heart monitors for people with cardiac disorders, the primary user category is relevant as a user may be measuring their own electrocardiogram (ECG) data, similarly a secondary user is also relevant as they may be measuring the ECG data of a child like Toby. Thus, passive users may also exist for these types of devices. While infrequent users are less common in this instance (as adverse events relating to the data collected are less likely to occur) there are still instances where adhoc users may be required to intervene. For example, if a wearer of the device has a medical event in a public setting their heart monitor may be indicating crucial information and whoever is first on scene may be asked to provide information to the emergency call handler which can be found on the device. The approach, therefore is relevant for different types of medical devices with different use cases, although the subset of users and specialist scenarios may differ.

Expert validation of the learnability matrix is required to further verify the extendibility of our approach to other medical devices but also to improve upon the existing framework. In future work, we recommend a team of usability experts with experience in medical systems and heuristic evaluation use the matrix independently to evaluate a medical device, with the aim to see how each expert interprets the matrix and the types of issues identified. Experts may compare to known learnability issues, such as those published by the Food and Drug Administration (FDA), and determine if the matrix identifies real world problems for each category of user considered. Furthermore, extending the evaluation to include severity ratings for the heuristic violations would enable quantitative scoring and assist in prioritisation of the design changes. This would result in a validated, practitioner-ready tool which can be used by industry and could be aligned with regulatory standards.

## 8 Conclusion

In this paper we present a methodology for evaluating the learnability of medical devices for different users in a variety of contexts of use. We define key categories of users, namely: primary, secondary, passive, infrequent, and adhoc users; and associated personas to investigate systems from different user perspectives. Using these categories and an existing set of heuristics for medical devices, we present a matrix which defines whether learnability is covered, partially covered or not covered by each heuristic enabling a secondary analysis which determines learnability issues in devices. This methodology is demonstrated using the MiniMed IP where real learnability issues are identified for each of the different user categories, highlighting the importance of considering different user perspectives. This enables evaluation of learnability for medical devices in a variety of contexts, but most importantly during safety-critical or adverse events which would not be ethical to investigate during typical user studies.

In future work, the learnability matrix can be validated through further analysis, using usability experts to determine if the majority of learnability errors are detected. Furthermore, the matrix can be applied to a variety of medical devices and contexts to determine the need for further user categories. This will help to refine the methodology and ensure its broader applicability in improving the learnability and safety of medical devices across diverse user groups.

## CRediT authorship contribution statement

**Jessica Turner:** Writing – review & editing, Writing – original draft, Visualization, Validation, Supervision, Software, Resources, Project administration, Methodology, Investigation, Funding acquisition, Formal analysis, Data curation, Conceptualization. **Judy Bowen:** Writing – review & editing, Writing – original draft, Visualization, Validation, Supervision, Software, Resources, Project administration, Methodology, Investigation, Funding acquisition, Formal analysis, Data curation, Conceptualization. **Veela Moxham-Bettridge:** Software, Investigation.

## Declaration of Competing Interest

The authors have declared no conflict of interest.

## Appendix A Supplementary material

MMC The supplementary materials contain the full learnability analysis for each of the tasks outlined above. The violations and adherences for the heuristic evaluation are presented including the secondary analysis for each user category. MMC

## Footnotes

1 See https://www.medtronicdiabetes.com/products/minimed-770g-insulin-pump-system.

2 See https://au.medtronicvirtualpump.com/2J2PfTbV5nNEgsMu/VirtualDemoPump/MiniMed_770G_OUS/gs3/mmol_l/.

3 See https://www.medtronicdiabetes.com/download-library/minimed-770g-system.
